# Toxic metals and genetic polymorphism in indigenous populations of northern Asia and America

**DOI:** 10.18699/vjgb-26-50

**Published:** 2026-05

**Authors:** B.A. Malyarchuk, N.V. Pokhilyuk

**Affiliations:** Institute of Biological Problems of the North of the Far Eastern Branch of the Russian Academy of Sciences, Magadan, Russia; Institute of Biological Problems of the North of the Far Eastern Branch of the Russian Academy of Sciences, Magadan, Russia

**Keywords:** genetic markers, genetic differentiation, human populations, Siberia, mercury, lead, cadmium, arsenic, генетические маркеры, генетическая дифференциация, популяции человека, Сибирь, ртуть, свинец, кадмий, мышьяк

## Abstract

The polymorphism of genes encoding enzymes involved in heavy metal metabolism was analyzed in indigenous Siberian populations based on the mercury, lead, and cadmium contents in the blood of Canadian Inuit carrying different genotypes. Additionally, we examined the polymorphism of genetic loci associated with sensitivity to arsenic exposure in indigenous Siberian populations using data on inorganic arsenic content in the urine of indigenous Andean populations who had consumed drinking water with elevated arsenic levels for thousands of years. A population genetic approach was used to seek genetic markers of toxic metal exposure in humans by analyzing genetic differences between populations living in different natural environments and under different conditions of toxic element contamination. Statistically significant differences were primarily observed between indigenous populations in Northeast Siberia (Siberian Eskimo (Yupik), Chukchi, and Koryaks) and samples from the central (Evens, Evenki, and Yakuts) and southern (Altaians, Shors, and Buryats) regions of Siberia. The maximum population branch statistics (PBS) values, which indicate the probable effect of selection on genetic loci sensitive to mercury exposure, were identified in seven gene loci: MTHFR (rs2274976 and rs1801131), GPX4 (rs713041), ABCB1 (rs1128503), AHR (rs2066853), TXNRD2 (rs5748469), and SEPHS2 (rs1133238). Loci rs713041 (GPX4), rs7483 (GSTM3), and rs2282143 (SLC22A1) can be considered genetic markers of lead exposure. Loci rs2274976 (MTHFR) and rs1056836 (CYP1B1) provide information about cadmium distribution in blood. It was found that protective variants of the AS3MT gene polymorphism are widespread (65.8 %) in the indigenous populations of Northeast Siberia. This is despite the lack of information regarding the long-term consumption of arsenic-contaminated drinking water by indigenous peoples along the Chukotka and Priokhotye coasts. It is hypothesized that seafood, which constitutes the core of the traditional “Arctic” diet of the indigenous populations inhabiting the coastal regions of the northern seas, may potentially be a significant source of arsenic and other toxic elements in Northeast Siberia. Further molecular, biochemical, and toxicological studies are necessary to elucidate the mechanisms by which toxic metals impact the genetic structure of indigenous populations in the Far North over long periods of time.

## Abstract (ru)

В настоящей работе у коренного населения Сибири проанализирован полиморфизм генов, кодирующих ферменты метаболизма тяжелых металлов, на основе данных о содержании ртути, свинца и кадмия в крови у канадских эскимосов – носителей различных генотипов; изучен также полиморфизм генетических локусов, связанных с чувствительностью к воздействию мышьяка по данным о содержании неорганического мышьяка в моче у коренного населения Анд, длительное время (в течение тысячелетий) потребляющего питьевую воду с повышенным содержанием мышьяка. Для поиска генетических маркеров воздействия токсичных металлов на организм человека использован популяционно-генетический подход, направленный на анализ генетических различий между популяциями, проживающими в разных условиях природной среды (и в разных условиях загрязнения токсичными элементами). Статистически значимые различия главным образом обнаружены между коренным населением Северо-Восточной Сибири (эскимосы, чукчи и коряки) и выборками из центральной (эвены, эвенки, якуты) и южной (алтайцы, шорцы, буряты) частей Сибири. Максимальные значения статистики PBS (population branch statistics), свидетельствующие о вероятном действии отбора на генетические локусы чувствительности к воздействию ртути, выявлены в семи локусах генов MTHFR (rs2274976 и rs1801131), GPX4 (rs713041), ABCB1 (rs1128503), AHR (rs2066853), TXNRD2 (rs5748469) и SEPHS2 (rs1133238). К генетическим маркерам воздействия свинца могут быть отнесены локусы rs713041 (GPX4), rs7483 (GSTM3) и rs2282143 (SLC22A1). В отношении распределения кадмия в крови информативны локусы rs2274976 (MTHFR) и rs1056836 (CYP1B1). Обнаружено, что в популяциях коренного населения Северо-Востока Сибири с высокой частотой (65.8 %) распространены варианты полиморфизма гена AS3MT, защищающие от воздействия неорганического мышьяка, хотя сведения о долговременном употреблении коренными жителями побережий Чукотки и Приохотья питьевой воды, загрязненной мышьяком, отсутствуют. Предполагается, что вероятным источником мышьяка, а также других токсичных элементов на Северо-Востоке Сибири могут быть морепродукты, являющиеся основой традиционной «арктической» диеты коренного населения прибрежных районов северных морей. Для прояснения механизмов долговременного воздействия токсичных металлов на генетическую структуру популяций коренного населения Крайнего Севера необходимы дальнейшие молекулярно-генетические, биохимические и токсикологические исследования.

## Introduction

Toxic metals such as mercury, lead, cadmium, and arsenic (a semimetal) are among the most widespread environmental pollutants. They are also toxic to humans and pose a health threat. The pollution of ecosystems in the Far North is of particular concern. As shown in a number of studies, various pollutants accumulate there and enter the human body through water, food, and air (Fernandez-Llamazares et al., 2020; Basu et al., 2022; Adlard et al., 2024). The traditional diets of indigenous peoples in the far northern regions of Asia and America, particularly in coastal areas, consist of foods (e. g., marine mammals, fish, and shellfish) that contain high levels of contaminants (Becker, 2000; Gamov et al., 2022). The industrial regions of Siberia also face the problem of heavy metal pollution, which endangers human health.

Despite the ongoing biomonitoring of pollutants in human biological tissues, primarily hair and blood, the interpretation of its data may be complicated by genetic differences between human populations living in different natural and climatic zones. Meanwhile, few population genetic studies are being conducted to identify genetic markers indicative of the impact of pollutants on the body. The Inuit of Canada and Greenland are the most studied populations in this regard (Ayotte et al., 2011; Ghisari et al., 2013; Parajuli et al., 2018, 2021).

The obtained data suggest that blood pollutant levels are associated with polymorphisms in genetic loci for several biological pathways, including folic acid, lipid, and xenobiotic metabolism. For instance, genetic variants linked to increased or decreased concentrations of heavy metals (mercury, cadmium, and lead) and organic pollutants (4,4′-DDE and PCB-153) in the blood have been identified in Canadian Inuit populations (Parajuli et al., 2018, 2021). It has been established that the indigenous populations of the Andes in Argentina, Chile, and Bolivia have genetically adapted to the toxic effects of drinking water with high concentrations of arsenic over thousands of years (Engström et al., 2011; Apata et al., 2017; De Loma et al., 2022). As a result, these populations have experienced a significant increase in the frequency of protective variants that encode enzymes metabolizing and eliminating arsenic from the body more efficiently.

In light of this, studies of the distribution of genetic va- riants related to the metabolism of toxic metals in other ethnic groups are relevant. Here we analyze the frequency distribution of polymorphic variants of genes involved in toxic element metabolism and associated with sensitivity to mercury, arsenic, cadmium, and lead in regional groups of indigenous Siberians

## Materials and methods

The study used the previously published results of the genotyping of 200 indigenous Siberian individuals, which had been obtained using the Illumina OmniExpress Bead Chips marker panel (Cardona et al., 2014). The northeast Siberian indigenous population sample (N = 58) included residents of the Bering Sea coast (Eskimo (Yupik) and Chukchi) and the Sea of Okhotsk coast (Koryaks). The Central Siberian sample included Evens from the Magadan Region, Evenks from the Krasnoyarsk Krai, and Yakuts from various regions of Yakutia (N = 70). The Southern Siberian sample included Shors from the Kemerovo Region, Southern Altaians from the Altai Republic and the Kemerovo Region, and Buryats from various regions of Buryatia (N = 72). Despite coexisting in the Severo-Evensk District of the Magadan Region since approximately the 16th century, the Koryaks and Evens are genetically distinct and classified into different regional groups (Cardona et al., 2014).

We examined indigenous Siberian populations, analyzing polymorphism in 78 loci presumably associated with interindividual differences in heavy metal (mercury, cadmium, and lead) concentrations in the blood of Canadian Inuit (Parajuli et al., 2018, 2021). The results are shown in Table S1^1^. These loci are located in genes that encode enzymes involved in key biological pathways, including antioxidant properties, folic acid metabolism, glutathione metabolism, hemoglobin, ion transporters, oxidative stress, selenoproteins, xenobiotic metabolism, metal-sensitive mechanisms (metallothioneins), lipid metabolism, and inflammatory processes.

Supplementary Materials are available in the online version of the paper: https://vavilovj-icg.ru/download/pict-2026-30/appx26.xlsx


Allele and genotype distribution, heterozygosity, and genetic differentiation of populations (Fst differences) were studied using the Arlequin 3.5 software package (Excoffier, Lischer, 2010). Deviations from the Hardy–Weinberg equilibrium in populations and differences in allele and genotype frequencies were assessed using Fisher’s exact test. The degree of linkage disequilibrium between alleles at two different loci on a chromosome was determined using r2 statistics. To assess the effect of selection on autosomal loci, the Population Branch Statistics (PBS) was used. This statistic is based on Fst differences between three compared populations (Yi et al., 2010). High PBS values may point at strong selective pressure on the locus, causing allele frequencies to change faster than would be expected by drift alone.

## Results and discussion

**Genetic markers of mercury exposure**

Mercury (Hg) is a toxic chemical element that threatens the health of people around the world. The high toxicity of inorganic and organic mercury compounds, especially methylmercury, is due to their interaction with thiol (-SH) groups of cysteine amino acid residues. This interaction causes disturbances in protein structure and enzyme active centers, as well as damage to cell membranes and organelles, resulting in increased oxidative stress (Arefieva et al., 2010; Basu et al., 2022).

Analysis of polymorphisms in loci associated with mercury metabolism in Canadian Inuit (according to data from R.P. Parajuli et al. (2018; 2021)) revealed statistically significant differences in allele distribution in 47 of 78 loci in pairwise comparisons of regional groups in Siberia (Table S1). Polymorphic variants of the seven loci with the highest PBS values, indicative of a probable selective effect on the locus (ranging from 0.266 to 0.043), had previously been demonstrated to be associated with blood mercury levels in Canadian Inuit carrying different genotypes (Table 1). These are loci rs2274976 (MTHFR gene), rs713041 (GPX4), rs1801131 (MTHFR), rs1128503 (ABCB1), rs2066853 (AHR), rs5748469 (TXNRD2), and rs1133238 (SEPHS2). The results of the analysis point to an increase in the frequency of alleles associated with elevated blood mercury levels from South to Northeast Siberia (Table S2). The highest frequencies of marker alleles (rs2274976-T, rs713041-C, rs1801131-G, rs1128503-A, rs2066853-G) were recorded among both Canadian Inuit and the indigenous populations of the coastal regions of Northeast Siberia (the Yupik, Chukchi, and Koryak peoples).

**Table 1.**
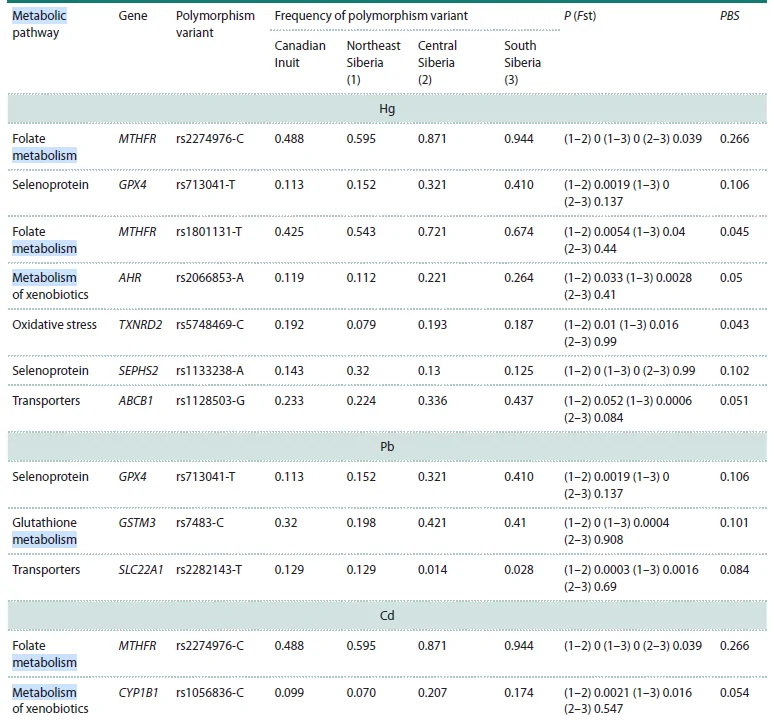
Distribution of polymorphism variants associated with sensitivity to heavy metal exposure among the indigenous populations of Siberia and Canadian Inuit Note. The data for the Canadian Inuit were obtained from R.P. Parajuli et al. (2018, 2021). P (Fst) is the statistical significance of Fst differences in allele frequency between Siberian populations in pairwise comparisons.

Additionally, the rs5748469-A variant of TXNRD2 and the rs1133238-A variant of SEPHS2 were only identified at maximum frequency in Northeast Siberia. Furthermore, the rs1133238-A allele primarily occurred in heterozygotes, who had the highest mercury concentrations in their blood. In terms of GG and GA+AA frequency, the Northeast Siberian sample significantly differs from other Siberian samples (P < 10–4, Fisher’s exact test).

The genetic loci identified as being associated with changes in blood mercury levels in Canadian Inuit are related to the following biological pathways: folate metabolism (MTHFR gene), selenoprotein metabolism (GPX4 and SEPHS2), transport of various molecules (ABCB1), xenobiotic metabolism (AHR), and oxidative stress (TXNRD2). According to ClinVar Miner (https://clinvarminer.genetics.utah.edu/), the polymorphism of the MTHFR gene (rs2274976, Arg594Gln) is functionally neutral, although a slight increase in blood homocysteine concentration was observed in heterozygotes (Melo et al., 2006). The MTHFR gene encodes methylenetetrahydrofolate reductase, which converts homocysteine into the biologically active form of folic acid.

Similarly, the polymorphism of the rs1801131 locus (Glu429Ala) leads to a slight decrease in the activity of the encoded enzyme but does not affect homocysteine levels in blood plasma. It is also considered a neutral change (Weisberg et al., 2001). Conversely, the Ala222Val substitution at the rs1801133 locus is associated with a significant decrease in MTHFR activity (Frosst et al., 1995). This results in an increased risk of hyperhomocysteinemia due to methylenetetrahydrofolate reductase deficiency. It should be noted that the indigenous populations of Northeast and Central Siberia have a lower frequency of the rs1801133-A variant, associated with lower enzyme activity, than other regions of the world (Table S1). The MTHFR gene has a block organization (Trifonova et al., 2012). In particular, the polymorphism variants at loci rs2274976 and rs1801131, located at a distance of 3.5 thousand base pairs (kbp) from each other, are in a state of linkage.

Our analysis demonstrated that the indigenous population of Northeast Siberia exhibited significantly higher linkage disequilibrium between these loci (r2 values ranging from 0.6 in the Chukchi to 1.0 in the Yupik) than more southerly populations (r2 values ranging from 0.2 to 0.3). Therefore, the elevated frequencies of the rs2274976-T and rs1801131-G variants and pronounced linkage disequilibrium in the indigenous northeastern Siberians may be of functional significance, potentially linked to alterations in methylenetetrahydrofolate reductase activity.

The rs713041-C variant of the glutathione peroxidase GPX4 gene, whose frequency increases to more than 80 % in northern Asia and America, appears to be important in protecting the body from the effects of peroxides, which are formed when exposed to various factors, including mercury. Studies have shown that GPX4 expression is higher for transgenes containing the rs713041-C variant when selenium levels are sufficient (Gautrey et al., 2011).

The SEPHS2 gene encodes selenophosphate synthetase 2, responsible for selenium metabolism, which in turn plays an important role in protecting against the toxic effects of mercury and methylmercury (Jorge et al., 2024). Thus, the association of the rs1133238-A variant with elevated blood mercury concentrations observed in Canadian Inuit may be related to the protective significance of this allele. The ABCB1 gene encodes P-glycoprotein, a transporter that removes various substances from cells, possibly including mercury (Sánchez Rodríguez et al., 2020). Therefore, the rs1128503-A variant, which is highly prevalent (approximately 80 %) in the Far North, may offer protection by reducing the toxic effects of mercury.

The AHR gene encodes the aromatic hydrocarbon receptor, which regulates the transcription of genes for enzymes that promote the metabolism of xenobiotics, including mercury, cadmium, arsenic, and nickel (Mohammadi-Bardbori et al., 2015; Alqahtani et al., 2024). The TXNRD2 gene encodes mitochondrial thioredoxin reductase 2, an enzyme involved in the antioxidant defense system. Polymorphisms in these genes appear to influence susceptibility to mercury poisoning (Crespo-Lopez et al., 2023).

**Genetic markers of lead exposure**

The main mechanism of lead (Pb) toxicity involves the inhibition of enzymes by binding to thiol groups of cysteine residues (Gonzalez-Villalva et al., 2025). There is no defined safety threshold for lead in the blood because even low concentrations of this chemical element can cause serious problems, such as damage to cell membranes, impaired hemoglobin synthesis, and oxidative stress. Studies of Canadian Inuit populations have revealed that their susceptibility to lead exposure is linked to several genes involved in selenoprotein metabolism (GPX4 gene), oxidative stress (TXNRD2), transport of various molecules (SLC22A1 and SLCO1B1), xenobiotic metabolism (CYP2C19), and other pathways (Parajuli et al., 2018, 2021).

Among indigenous Siberian populations, the greatest differentiation (PBS values ranging from 0.106 to 0.08) was found between the northeastern Siberian sample and other groups at loci rs713041 (GPX4), rs7483 (GSTM3), and rs2282143 (SLC22A1) (Tables S3 and S4). Thus, the rs713041 locus of the GPX4 gene for glutathione peroxidase appears to protect the body not only from mercury exposure but also from lead toxicity (Table 1).

The GSTM3 gene encodes glutathione S-transferase mu 3, which plays an important role in the detoxification of xenobiotics. The rs7483-T variant has been shown to significantly increase the activity of this enzyme by leading to the amino acid substitution Val224Ile, allowing for more efficient metabolism of drugs (Tetlow et al., 2004). This increased activity may also affect the metabolism of heavy metals, particularly lead. Therefore, the increased frequency of the rs7483-T variant of the gene, which encodes the more active form of GSTM3, among the indigenous northeastern Siberians, compared to other regional groups in Siberia and Canadian Inuit, is apparently associated with its protective role against the toxic effects of lead.

The SLC22A1 gene encodes organic cation transporter 1 (OCT1), which plays an important role in transporting various substances, including drugs, across cell membranes. The rs2282143-T variant has been shown to reduce the ability of OCT1 to transport drugs into cells without significantly affecting SLC22A1 gene expression (Chen et al., 2018). Therefore, the increased frequency of the rs2282143-T allele in indigenous populations of Northeast Siberia and Canadian Inuit may be associated with the protective role of this genetic polymorphism variant in response to heavy metal exposure.

The ALAD gene is the best known gene associated with increased sensitivity to lead poisoning (Stajnko et al., 2024). This gene encodes δ-aminolevulinic acid dehydratase (ALK dehydratase), which plays a role in the synthesis of porphobilinogen, the precursor of tetrapyrroles (heme and other compounds). The ALK dehydratase enzyme is highly sensitive to inhibition by heavy metals (Perini et al., 2024). Polymorphism at the rs1805313 locus has been shown to be closely associated with blood lead levels and to influence the expression of ALK dehydratase in blood cells (Warrington et al., 2015). Although no differences in blood lead and mercury concentrations were found among Canadian Inuit with different rs1805313 genotypes, comparative analysis revealed increased frequencies of the rs1805313-A variant in Canadian Inuit and indigenous peoples of Northeast Siberia (PBS value = 0.143) (Table S4). This may be due to the protective role of this ALAD gene polymorphism against lead.

**Genetic markers of cadmium exposure**

Cadmium (Cd) is one of the most common environmental pollutants, and it exerts several toxic effects. These include changes in gene expression, inhibition of DNA repair processes, interference with apoptosis and autophagy, oxidative stress, and interaction with trace elements necessary for the catalytic activity of enzymes (Qu, Zheng, 2024; Wang et al., 2025). Cadmium can compete with trace elements (e. g., Zn, Mn, Fe, Se, and Mg) for binding sites in the active centers of antioxidant enzymes, damaging them in the process (ĐukićĆosić et al., 2020).

For people living in Arctic regions, the main sources of cadmium exposure are not only the consumption of traditional foods but also smoking (Becker, 2000; Adlard et al., 2024). For instance, Canadian Inuit individuals have been found to have blood cadmium concentrations eight times higher than normal (Parajuli et al., 2021). They also identified associations between cadmium levels and polymorphisms in genes associated with antioxidants (TXNRD2 and SELS), oxidative stress (NOS1), transport (ABCC1), cytochrome P450 (CYP2D6 and CYP24A1), and folate metabolism (MTHFR). We analyzed the distribution of polymorphism variants at several loci associated with cadmium metabolism in indigenous Siberians. However, the analysis revealed only one variant of the MTHFR gene, rs2274976-T. A statistically significant increase in the frequency of this variant is observed in both Northeast Siberia and among Canadian Inuit (Table 1 and Table S5). Among the latter group, the increased frequency of the rs2274976-T allele is accompanied by increased blood concentrations of cadmium and mercury, as noted above.

Statistically significant differences between the sample from Northeast Siberia and other Siberian groups were also found in the distribution of alleles of the rs1056836 locus of the CYP1B1 gene. This locus also proved to be associated with differences in blood cadmium distribution among Canadian Inuit (Table 1 and Table S2). The rs1056836-G variant frequency is highest (over 90 %) among indigenous peoples in Northeast Siberia and the Canadian Inuit. This variant is associated with lower blood cadmium levels.

Also, our research identified genetic loci potentially associated with heavy metal exposure. These loci are characterized by relatively high PBS (ranging from 0.087 to 0.043). Statistically significant increases in allele frequencies were found in Canadian Inuit and indigenous peoples of Northeast Siberia for cadmium (rs688 of the LDLR gene) and mercury (rs628031 of the SLC22A1 gene and rs732774 of the ATP7B gene), and only in northeastern Siberians for mercury (rs4895441 of the HBS1L gene) (Table S6). However, no differences in heavy metal content were found among Canadian Inuit carriers of different genotypes at these loci. Nevertheless, these loci are promising for further research in the field of environmental genetics of indigenous Siberian populations.

**Genetic markers of arsenic exposure**

Arsenic (As) is an element that can pose a threat to human health. Most seafood has elevated levels of arsenic. However, marine animals such as whales and seals mainly contain arsenic in the form of arsenobetaine, which appears to be nontoxic and harmless to humans (Becker, 2000). Nevertheless, small amounts of other organic forms of arsenic are present in seafood and may be more toxic than inorganic arsenic (Bagryantseva, Khotimchenko, 2021). It has been shown that organic forms of arsenic can be converted into methylated and inorganic forms during metabolism. These forms can damage biological molecules and induce negative effects in metabolic processes (Bagryantseva, Khotimchenko, 2021).

Previous studies indicated that long-term consumption of water with high arsenic concentrations led to the selection of genetic variants that promote arsenic detoxification in indigenous Andean populations (Schläwicke Engström et al., 2009; Eichstaedt et al., 2015; Apata et al., 2017; De Loma et al., 2022). First, these changes affect the AS3MT gene for arsenic methyltransferase, which converts inorganic arsenic (iAs) into monomethylarsonic acid (MMA) and less toxic dimethylarsinic acid (DMA).

The activity level of AS3MT can be assessed from the ratio between DMA and MMA in urine: the more active the enzyme, the lower the percentage of MMA and the higher the percentage of DMA. Studies of female residents of San Antonio de los Cobres (SAC) in northern Argentina who consume water with a high concentration of arsenic (approximately 0.2 mg/L) have shown that other enzymes besides arsenic methyltransferase are involved in arsenic detoxification. These enzymes include those involved in one-carbon metabolism (MTRR and CHDH genes) and antioxidant protection (PRDX2 and GLRX genes) (Schläwicke Engström et al., 2009).

To date, a number of mutations in the AS3MT gene have been identified that affect the enzymatic efficiency of different forms of arsenic methyltransferase. We examined the population distribution of polymorphism frequencies at seven AS3MT gene loci and nine loci in the CYP17A1, PRDX2, DNMT1a, INMT, MTRR, CNNM2, WBP1L, and N6AMT1 genes (Table S7). Statistically significant differences and maximum PBS values ranging from 0.217 to 0.083 were identified between indigenous populations of Northeast Siberia and other regions at the AS3MT gene loci rs7085104 and rs10786719 and the CYP17A1 gene loci rs743572 and rs17115100.

It has been shown that a widespread protective haplotype is prevalent among Argentinians and Bolivians as a result of their adaptation to elevated levels of arsenic in their drinking water. Allelic variants of this haplotype are associated with the lowest MMA levels in urine (De Loma et al., 2022). One of the marker variants of this haplotype is rs7085104-G, which occurs at a frequency of 72.9 % in Argentinians (Engström et al., 2011). Our analysis of AS3MT and CYP17A1 gene polymorphisms in the indigenous Siberian population also demonstrated close linkage between polymorphism variants at the rs743572, rs7085104, and rs10786719 loci, which are separated by 40.8 kbp (see the Figure).

**Fig. 1.**
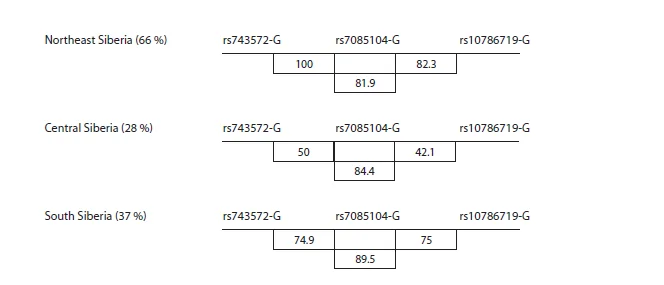
Linkage disequilibrium (r2) between polymorphism variants at loci rs743572 of the CYP17A1 gene and rs7085104 and rs10786719 of the AS3MT gene in the indigenous population of Siberia.

The rs7085104-G variant had the highest frequency (65.8 %) and the GGG haplotype was most prevalent in northeastern Siberians (Table 2). Thus, the results show that the frequency of the polymorphic AS3MT and CYP17A1 variants sensitive to the effects of arsenic increases in the northeastern direction in indigenous Siberian populations

**Table 2.**
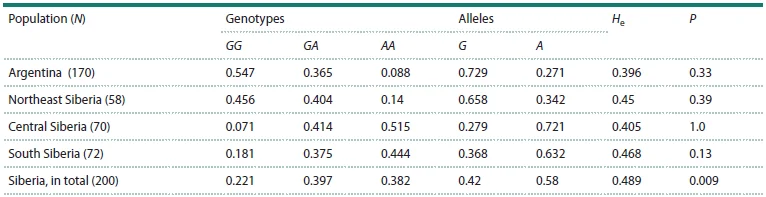
Frequencies of genotypes and alleles of locus rs7085104 of the AS3MT gene in indigenous populations of Siberia and Argentina Note. N – sample size. He – expected heterozygosity; P – statistical significance of deviation from Hardy–Weinberg equilibrium (significant at P <0.05). Data for the Argentine sample are taken from (Engström et al., 2011).

The main cause of this trend seems to be the traditional diets of indigenous peoples in the coastal regions of Northeast Siberia, such as the Eskimo, Chukchi, and Koryaks. Their ancestors have long consumed seafood (fish, shellfish, algae, and marine mammals), and the indigenous peoples of the northern seas still focus their diets on seafood. However, it is known that seafood contains high concentrations of heavy metals and arsenic (Becker, 2000; Gamov et al., 2022).

Arsenic in such products is primarily found in an organic form that is less toxic (mainly as arsenobetaine and arsenocholine), though it is also present in small amounts in an inorganic form (e. g., in animal liver and kidneys) (Bagryantseva, Khotimchenko, 2021). Constant seafood consumption over generations could have led to the selection of AS3MT variants that allow for more efficient elimination of iAs. Interestingly, our results showed that the optimal variants of genetic polymorphism in Northeast Siberia were the same as those found in indigenous Andean populations, who had consumed water with elevated concentrations of inorganic arsenic for a long time.

## Conclusion

This study analyzes genetic polymorphisms associated with sensitivity to toxic metals (mercury, lead, cadmium, and arsenic) in indigenous Siberian populations. Statistically significant differences were primarily observed between populations in Northeast Siberia (Eskimo (Yupiks), Chukchi, and Koryaks) and those in Central and Southern Siberia. The maximum PBS values indicating the probable effect of selection on genetic loci sensitive to mercury exposure were identified in seven gene loci: MTHFR (rs2274976 and rs1801131), GPX4 (rs713041), ABCB1 (rs1128503), AHR (rs2066853), TXNRD2 (rs5748469), and SEPHS2 (rs1133238). Loci rs713041 (GPX4), rs7483 (GSTM3), and rs2282143 (SLC22A1) can be considered genetic markers of lead exposure. Loci rs2274976-T (MTHFR) and rs1056836 (CYP1B1) may provide insight into cadmium distribution in the blood.

It should be noted that, in most cases, indigenous Siberian populations show an increase in genetic variants associated with elevated levels of mercury, lead, and cadmium in the northeastern direction. Furthermore, this trend aligns with the findings regarding Canadian Inuit populations (Parajuli et al., 2018, 2021). In our opinion, the most likely reason for this is the long-term adaptation of indigenous peoples along the Bering, Chukchi, and Okhotsk Sea coasts to their specific natural environment. This adaptation occurred over several millennia, and it is mainly due to their high seafood consumption. Seafood is rich in beneficial micronutrients, omega-3 polyunsaturated fatty acids, and selenium. However, it also contains toxic substances.

The widespread occurrence of genetic variants associated with elevated levels of heavy metals in the blood among Eskimo, Chukchi, and Koryaks is likely due to the formation of relatively harmless complexes of heavy metals with selenium and other detoxifying agents, such as glutathione and thioredoxin. These complexes reduce the bioavailability of metals and minimize their harmful effects. However, additional molecular-genetic and biochemical studies of indigenous populations are needed to determine the true causes. The available literature on the mechanisms of heavy metal detoxification and the risks of diseases caused by exposure in indigenous peoples of the Far North is insufficient (Ayotte et al., 2011; Ghisari et al., 2013; Parajuli et al., 2018, 2021).

The genetic consequences of human adaptation to environmental exposure to inorganic arsenic appear to be a betterstudied issue (González-Martínez et al., 2024). Studies of indigenous populations in mountainous regions of Argentina, Chile, and Bolivia have shown that long-term consumption of drinking water with elevated arsenic levels has resulted in the selection of AS3MT gene polymorphism variants that enable more efficient arsenic methylation and elimination (Schlebusch et al., 2015).

Our study shows that the prevalence of AS3MT gene polymorphisms that protect against exposure to inorganic arsenic is significantly higher in indigenous northeastern Siberians. However, we have no information about long-term consumption of arsenic-contaminated drinking water by indigenous inhabitants of the Chukotka and Priokhotye coasts. Seafood is likely a source of arsenic in Northeast Siberia because it contains organic and inorganic arsenic. Further molecular-genetic and biochemical studies of indigenous Far North populations, as well as the microelement composition of groundwater and traditional food sources, are necessary in this regard.

## Conflict of interest

The authors declare no conflict of interest.
